# Regulating with food: a qualitative study of Neurodivergent experiences in adults with binge eating disorder

**DOI:** 10.1186/s40337-025-01493-7

**Published:** 2025-12-09

**Authors:** Lauren Makin, Adia Meyer, Valeria Mondelli, Kate Tchanturia

**Affiliations:** 1 PO59, Department of Psychological Medicine, Institute of Psychiatry, Psychology & Neuroscience, King’s College London, 16 De Crespigny Park, London, SE5 8AF UK; 2 National Institute for Health and Care Research (NIHR) Maudsley Biomedical Research Centre at South London and Maudsley NHS Foundation Trust and King’s College London, London, UK; 3 Department of Eating Disorders, South London and Maudsley NHS foundation Trust, London, UK; 4 Department of Psychology, Ilia State University, Tbilisi, Georgia

**Keywords:** Binge eating, Autism, ADHD, Neurodivergence, Lived experience, Eating disorders, Qualitative research, Treatment, Clinical, Thematic analysis

## Abstract

**Background:**

ADHD and Autism are overrepresented in adults with binge eating disorder (BED) and are linked to unique mechanisms and poorer treatment outcomes. This qualitative study explored how Neurodivergent individuals with BED understand their Neurodivergence in relation to their BED and treatment needs.

**Methods:**

Ten Neurodivergent adults (or those who score above threshold on screeners; AQ-10 > 5, ASRS-Screener > 3) who exhibited binge-type eating pathology were interviewed. The data were analysed with reflexive thematic analysis.

**Results:**

We generated four themes: the need for greater self-understanding of the Neurodivergence–BED link; Neurodivergent-related factors contributing to irregular eating; Bingeing to regulate Neurodivergent-related sensory, stimulation, and emotional needs; and the importance of personalised, adaptable support that accounts for cognitive, sensory, and communication differences.

**Conclusions:**

These findings suggest that ADHD and Autism can influence BED experiences and understanding this can improve treatment engagement and guide the development of more effective personalised treatments for Neurodivergent individuals. Furthermore, these findings allow us to expand existing models of how Neurodivergence interact with eating disorders to include BED.

## Abstract (plain language summary)

Neurodivergence, such as Autism and ADHD, is more common in people with binge eating disorder, which involves repeatedly eating large amounts of food while feeling unable to stop, often followed by guilt or distress. Little is known about how Neurodivergence affects this eating disorder or what treatments work best. We spoke to ten adults with binge eating disorder who had diagnoses or traits of Autism or ADHD. They described wanting help understanding how their Neurodivergence influenced their eating disorder. Factors like appetite-suppressing medication, missing hunger cues due to distraction, or irregular routines made bingeing more likely. Participants also used bingeing for comfort, reward, or seeking certain textures or tastes. Participants wanted treatment that was personalised, flexible, and understanding of Neurodivergent needs. Our findings suggest psychoeducation about Neurodivergence and adapting therapies to address sensory, emotional, and routine-related challenges may improve treatment engagement and outcomes.

## Background

‘Neurodivergence’ is an umbrella term which describes neurotypes that differ from what is typically considered the societal ‘norm’ [1]. It stems from the Neurodiversity paradigm, which frames such differences as natural variations in human cognition rather than deficits [2]. ADHD and Autism are common forms of Neurodivergence that often co-occur (e.g. 50–70% of Autistic people are also ADHD [3]) and are associated with differences from neurotypical development in executive functioning, sensory processing, and communication [4, 5].

While the term Neurodivergence is sometimes used more broadly to include conditions like Dyslexia or OCD [1, 6, 7], ADHD and Autism are the neurotypes most associated with eating disorders (EDs) and have been the primary focus of research in this context [7, 8]. ADHD and Autism are overrepresented in adult ED populations, including those with binge eating disorder (BED), and are associated with distinct experiences, mechanisms, and treatment needs [9–11]. OCD is also more prevalent in EDs and is associated with more severe ED symptomatology and poorer treatment outcomes [7]. While we acknowledge that Neurodivergence involves a wide range of variations, we focus on Autism and ADHD for the purposes of this study.

BED is characterised by recurrent episodes of binge eating, during which individuals eat comparably large amounts of food while experiencing a loss of control and subsequent feelings of distress, shame, and guilt, without compensatory behaviours [4]. BED is commonly associated with higher weight and with somatic and mental health comorbidities [12]. Autistic traits, measured by a range of screeners, are significantly elevated in individuals with BED compared to healthy controls [9]. Similarly, ADHD is significantly more prevalent in people with BED; 10% of adults with BED meet diagnostic criteria for ADHD [9], compared to 3% of the general population [13]. ADHD traits remain stable over time, regardless of recovery status and are more pronounced in BED than in restrictive anorexia nervosa (AN) [9].

### Perceived causal mechanisms

In qualitative studies on Neurodivergence and EDs, participants have suggested unique mechanisms underlying ED development and maintenance in Neurodivergent individuals, though most have focused on Autism and AN [7]. A recent systematic review and synthesis identified six qualitative studies exploring Autistic people’s experiences of AN [8], which reported a range of diverse perceived mechanisms linking Autistic traits with AN [14–19]. Exteroceptive hypersensitivity was thought to contribute to AN through aversive reactions to food textures, tastes, smells, temperature, colours, and overstimulating environments [14, 15, 17–19]. Interoceptive hyposensitivity, such as challenges recognising hunger, thirst and digestion, was also thought to lead to undereating [14, 15, 17–19]. Additionally, experiencing heightened emotions, overwhelm, and difficulties identifying, regulating, and communicating emotions, were perceived as linked to restrictive eating to numb or distract from these [14, 15, 18, 19]. Monotropism [20], a thinking style characterised by tending to focus on a small number of interests, often fostering deep expertise and preference for routine, was thought to promote strict food rules that could escalate into AN [14, 15, 17, 18].

Social and communication differences, especially with peers, were also linked to AN [14–19]. This likely stemmed from non-Autistic people’s reactions to these differences, including judgment, misunderstanding, neuro-normative expectations, and discrimination, which can cause significant emotional and psychosocial distress for Autistic people. Autistic people may then respond by masking (camouflaging), which in turn causes low self-esteem and identity or self-concept struggles [21–23]. Thus, a lack of social acceptance, accommodations, and the broader systemic discrimination faced by Autistic people may therefore contribute to AN development [7], with some individuals using weight control as a means of seeking acceptance and belonging [14, 15, 18, 19]. This risk is likely compounded by delays in Autism recognition and diagnosis [14, 15, 17–19, 24]. Supporting this, Brown and colleagues [25] found that while Autistic traits were associated with higher ED risk, the effect was notably reduced in women with a formal Autism diagnosis, suggesting that timely recognition may be protective.

Fewer studies have examined other forms of Neurodivergence or other EDs [9]. To date, only one study has focused on bulimia nervosa (BN), where Autistic traits were also linked to restrictive eating through sensory differences, monotropism, and social difficulties [26], paralleling findings from Autism-AN samples [14–19]. The same study also examined ADHD, identifying links between ADHD traits and binge eating [26]. Many of these ADHD traits mirrored those reported in the Autism-AN literature [14, 15, 17–19], including emotional, sensory, and thinking style differences, though some divergences were noted in the experience of sensory and social factors [26]. These findings suggest that shared mechanisms may operate across different forms of Neurodivergence and ED diagnoses. However, the literature remains dominated by Autism-AN studies [7], with only one on BN [26], and none on BED. Focusing on a BED sample, therefore, offers an opportunity to extend this transdiagnostic picture and identify both overlapping and distinct pathways between Neurodivergence and EDs.

### Treatment needs

Neurodivergent patients with EDs also likely have distinct treatment needs arising from their Neurodivergence, though again, most research has focused on Autism and AN. Autistic patients with EDs report poorer treatment experiences, higher psychosocial difficulties, and a greater risk of longer inpatient stays, partly due to limited autism-informed care [10]. In the meta-synthesis [8], Autistic patients with AN described feeling misunderstood by ED professionals [14, 17–19, 24, 27, 28], that clinicians are often uncertain about how to disentangle patients’ Autism from their AN [14, 17, 18], and disappointment with current psychological treatments [14, 17, 18]. Some even describe experiencing iatrogenic harm from standard approaches, including CBT, and yet harm assessment and reporting is still under-prioritised [29, 30]. Delayed Autism recognition, often identified years after ED diagnosis [14, 15, 18], was also seen as a barrier to earlier self-understanding and tailored support [15, 18, 19]. This is likely especially true for women, who face longer diagnostic delays for Autism and higher ED prevalence [31, 32].

Autistic patients with AN emphasised a desire for specific treatment adaptions, including: sensory adjustments to the environment and meal plans [14, 17, 18]; flexible communication options [14, 17–19]; awareness of different thinking styles like monotropism [14, 17, 18]; sufficient time to build trust and process information [17, 18]; teaching adaptive coping skills [14–18]; and support for carers [17, 18]. They also highlighted the benefits of increased clinician training in Autism [17, 18], avoiding a ‘one-size-fits-all’ approach [14, 17, 18], and only trying to change Neurodivergent behaviours that contribute to the AN, in which case interventions should be gradual, and neuro-affirmative [17, 18]. Self-determination is particularly important, as many treatments focus on compliance, which is not necessarily a good fit for Autistic people and can even be harmful [33].

Autistic patients also strongly support the use of the PEACE pathway [17], a series of Autism-informed adaptions to ED treatment which can be implemented into existing services [34–36]. The PEACE pathway includes staff training, environmental adjustments, communication passports, and sensory workshops [35, 37, 38]. This pathway received positive feedback from patients and clinicians and reduced lengths of admission for patients with AN and Autistic traits, saving approximately £22,837 per patient [39, 40].

Less is known about treatment needs in other Neurodivergent populations or EDs. ADHD patients with EDs have higher dropout rates and poorer treatment outcomes than non-ADHD peers [11], and ADHD adults with obesity exhibit poorer treatment outcomes from behavioural and pharmacotherapy weight loss interventions [41]. These patients also have increased complications during and after bariatric surgery [41]. ADHD and Autistic patients with BN reported similar treatment needs to those with AN, including support in developing a Neurodivergent identity [15, 18, 19], distinguishing Neurodivergent traits from those driving the ED [17, 18], and rejecting a ‘one-size-fits-all’ approach [14, 17, 18], while also emphasising the importance of structure and routine-building in recovery [26].

While important progress has been made in understanding the intersection of Autism and AN, to date, no studies have examined treatment needs in Neurodivergent patients with BED. Given that patients with BED are already underserved within ED services [42, 43], and that treatment models often prioritise restrictive EDs, it is essential to establish whether Neurodivergence-informed adaptions differ or overlap in BED.

### Current study

This study aimed to explore what Neurodivergent individuals with BED describe regarding their Neurodivergence and ED experiences. It was guided by two research questions: (1) *Perceived causal mechanisms -* How do Neurodivergent adults with BED perceive their Neurodivergence as influencing BED behaviours? And (2) *Treatment needs –* What are their self-identified treatment needs due to their Neurodivergence?

This study was motivated by feedback from the PEACE pathway [34], highlighting gaps in ADHD and binge-type ED care, and aligns with community-identified research priorities (understanding causal mechanisms and improving treatment outcomes) [44]. Our aim is to contribute to a more inclusive, person-centred understanding of BED among Neurodivergent individuals and to inform best practices for care.

## Materials and methods

### Study design

This qualitative study used semi-structured interviews and reflexive thematic analysis (RTA) to explore how Neurodivergent adults with experiences of BED understand their Neurodivergence, how their Neurodivergence influences their ED, and what support they need. NHS ethical approval was obtained (REC: 24/LO/0573).

A critical realist framework, informed by the Neurodiversity paradigm, underpinned the work [6, 45–47], recognising traits as real but their social meanings as constructed. Language choices (e.g. ‘Neurodivergent’, ‘Autistic’) reflect neuro-affirmative principles and community preferences [48–50], but quotations retain participants’ original wording, including deficit-based terms, to reflect their perspectives. This also acknowledges that some experiences of Neurodivergence may involve intrinsic challenges [1], though may also reflect internalisation of self-stigma.

### Participant recruitment

Participants were eligible if they were aged 18 or over; had a current or previous presentation of BED (clinically diagnosed or self-reported sub-clinical), and either self-identified as Neurodivergent, Autistic, or ADHD, self-reported a formal Autism or ADHD diagnoses, or scored above screening thresholds (ASRS-Screener >3; AQ-10 >5). We included self-identified or high-trait individuals due to diagnostic barriers and to align with current ED service practices [40, 51, 52].

Ten participants were interviewed. Six participants were recruited from the South London and Maudsley (SLaM) NHS Foundation Trust ED Service, which has implemented the PEACE pathway. All adult SLaM patients with a recorded BED diagnosis who had consented to be contacted for research were invited to participate, regardless of known or suspected Neurodivergence. Clinicians acted as gatekeepers using an opt-out approach. One participant withdrew, leaving six from this recruitment method. An additional three participants were recruited during educational events, and one joined through a concurrent SLaM survey. Patients received study materials via email, including the participant information sheet, consent form, and the ASRS-Screener and GSQ-14 (not analysed here).

### Data collection

Once the consent form and screeners were returned, clinical assessment data was extracted from SLaM’s ED database (*n* = 7). Non-SLaM participants completed an equivalent questionnaire (*n* = 3).

Interviews were conducted from February to June 2025 by LM and AM. In line with AASPIRE guidelines for Autistic inclusion [53], interviews were offered in-person or via video call, phone, or instant messaging. Most chose video (50%) or in-person (40%), and one chose via instant messaging (10%). At the beginning of the interview, participants were told if they screened positively on the ASRS-Screener or AQ-10, with clarification that these were not diagnostic tools. Participants could complete the interview in one or two sessions – all chose one. Interviews averaged 42 min (range = 31–59).

Participants received a £25 voucher for taking part in the interview. All participants also received the preliminary findings and were invited to share feedback (*member reflections)*. Two (20%) responded, saying they felt involved and comfortable in the research, valuing the opportunity to share experiences to help and educate others. Respondents reported that many findings resonated, though they noted that individual differences in experiences of Neurodivergence and EDs meant not all themes reflected every individual’s experiences.

### Measures

#### Adult ADHD self-report screener scale (ASRS-screener)

Used to screen for high ADHD traits. It includes six questions predictive of DSM-IV-TR criteria [54], scored 0–1. A total score of >3 indicates probable ADHD. It has demonstrated reliability in ED populations [55] and is widely used [11, 56–60]. In the current study, internal consistency was acceptable (*α* = 0.75), and eight participants scored above threshold. This may reflect sample heterogeneity, screener brevity, false positives, or sample bias (as ADHD was mentioned in study invites).

#### Autism spectrum quotient – 10 items (AQ-10)

Used to screen for high Autistic traits [61]. It consists of 10 items scored 0–1, with a clinical cut-off of >5, and appears to be equally sensitive to Autism in both women and men [62]. Though widely used in ED research, its reliability in acute settings is debated due to low specificity (28%) and internal consistency (*α* = 0.64) [63, 64]. Still, it is the only Autism screener currently recommended by NICE for adult clinical use. In the current study, internal consistency was questionable (*α* = 0.68).

#### Interview schedule

The interview was developed from previous research [10–12, 46] and covered ED development/maintenance and treatment experiences, referring explicitly to ADHD, Autism, and/or Neurodivergence. It was reviewed by the principal author, KT, who is an experienced clinician. After three interviews, the schedule was revised. Questions that did not elicit relevant data were removed, and additional questions based on findings so far were added.

### Data analysis

Interviews were audio-recorded, transcribed (either manually verbatim or auto-transcribed and checked), and anonymised. Full transcripts are available on request, including for secondary analysis, as specified in the participant consent form.

We used RTA to explore participants’ understanding of their Neurodivergence and ED, focusing on identity, causal mechanisms, and support needs. Initial coding was conducted by AM in NVivo, followed by sub-theme generation. Overarching themes were generated through diagramming on paper and then revisiting the data in NVivo. These were then discussed with LM, who acted as a *critical friend* to assess credibility, enhance reflexivity, and challenge assumptions [65]. Themes were then shared with participants for *member reflections* [65], addressing the double empathy problem – mutual misunderstandings that can arise between Neurodivergent and non-Neurodivergent individuals due to differing ways of experiencing the world [66]. Feedback was used to enhance analysis, rather than to ‘validate’ findings [65], and no participants reported feeling misrepresented.

### Positionality and reflexivity

LM is a PhD student in ED and Neurodivergence research, with a background in experimental psychology. Her approach shifted through the project toward critical realism. AM is a clinician in a group-based ED programme with training in social psychology and therapy. Her practitioner lens helped ground interpretations in clinical practice and informed ethical decisions, such as avoiding dual roles. We chose not to disclose fixed identities but privately reflected on how varying degrees of shared experience with participants shaped our interpretations.

We were also guided by previously identified lived experience priorities [44], incorporated long quotes to preserve participants’ voices, shared findings for feedback and inclusion, followed AASPIRE’s inclusive research guidelines [53], and framed the study within a critical Neurodiversity paradigm aimed at improving ED care for Neurodivergent individuals.

## Results

### Sample characteristics

Ten adults (aged 18–54 years; *M* = 35) participated in the study. Most participants identified as female (*n* = 9, 90%) and heterosexual (*n* = 7, 70%). The sample comprised four White, two Black, two Asian, and two mixed-ethnicity participants. Diagnoses included BED (*n* = 6), OSFED (*n* = 1), and subclinical binge-eating behaviours (n = 3). BMI data was available for six participants and ranged from ‘healthy weight’ to ‘obese’ (20.8–36.3).

Of the seven participants recruited through SLaM, four had completed treatment, two were currently receiving treatment, and one remained on the waiting list. In total, six SLaM participants and one non-SLaM participant had received ED treatment at the time of interview. Reported interventions were primarily self-guided help or CBT-T at SLaM, as well as CBT-E, CAT, inpatient treatment, schema therapy, various group therapies, and counselling.

Participants self-reported ADHD diagnoses (*n* = 3), suspected ADHD (*n =* 3), suspected Autism (*n =* 3), diagnosed Dyslexia (*n =* 1), suspected Dyslexia (*n =* 1), suspected OCD (*n =* 1; which they identified as Neurodivergence), and self-identified as ‘Neuro-spicy’ *(n* = 1). Eight participants screened above threshold on the ASRS-Screener (M = 5.0), and three scored above threshold on the AQ-10 (M = 4.7). Co-occurring self-reported conditions included anxiety (*n* = 3), depression (*n* = 2), borderline personality disorder (*n* = 1), PTSD (*n* = 1), functional neurological disorder (*n* = 1), and seizures (*n* = 1).

### RTA findings

**Fig. 1:**
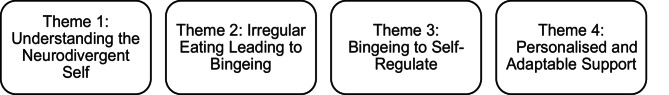
The four generated themes

Participants in this study discussed their experiences of Neurodivergence, BED, and ED treatment. Four themes were generated from this data (see Fig. 1).

### Theme 1: Understanding the Neurodivergent self

Many participants were unsure whether or how their Neurodivergence affected their BED, saying *“I don’t know”* [026] and *“I haven’t thought about it”* [022], and wished clinicians had explored this with them, saying *“because it’s something that hasn’t really been discussed or looked at*,* it’s not something that I’ll know what needs to be managed”* [009]. This was often exacerbated by participants being unsure if they were Neurodivergent at all, as they lacked formal diagnosis, saying “*there’s no way to*,* like*,* say*,* oh*,* it’s this or that. So*,* for now*,* it’s just*,* like*,* damn”* [009].

*“It didn’t come up in therapy because I hadn’t thought about it. But if they were to […] bring it up*,* I think it could have made things a lot more clearer for me. Now that we’re having a discussion*,* I’m like*,* oh*,* wow*,* like*,* there’s a lot of things that could be like*,* yeah*,* parallels. And there’s a lot of explanations that were applicable.”* [032].

Those with diagnosed ADHD often felt it contributed to binge eating, and understanding this link - especially by recognising triggers, underlying needs, and behaviour patterns - made prevention easier, saying *“once you understand the reasons that you do stuff*,* then it’s much*,* much easier to change your behaviour”* [002]. Social media, not professionals, was the main source of this insight, leading participants to wish such connections were discussed at diagnosis or in treatment, saying “*I think an outline of what it could look like*,* [the clinician saying] ‘you’ve got this diagnosis*,* you may struggle with food’*,* so it’s not like a dirty little secret”* [031]. Additionally, OCD was felt to perpetuate BED, saying *“I think it keeps me in a cycle”* [023].

*“I came across an account in 2020 that was talking about like ADHD and disordered eating and I think that was the first time that I recognised that that’s what it is”* [001].

### Theme 2: irregular eating leading to bingeing

ADHD-related factors, such as long-acting medication, hyperfocus, and interoceptive issues, meant participants forgot to eat or missed hunger signals. External prompts like timers, routines, or reminders from others helped.

*“I have an app*,* a fasting app. Which because I’m not purposely fasting*,* it kind of has alarms on me to enlighten you when you’ve done 24 hours when you’ve done 32 hours. So*,* it reminds me to actually eat.”* [031].

Food shopping and preparation felt *“overwhelming”* [001] due to sensory overload, energy demands, and decision fatigue, and so were often avoided, saying *“the idea of cooking for me is something that I find really daunting”* [032]. Strategies included ready-to-eat meals, frozen ingredients, ‘same fooding’, and sensory aids like headphones.

Strong food preferences, texture sensitivities, and fixation on specific foods also influenced eating patterns, sometimes leading to skipped meals, saying *“Sometimes you know I might skip lunch there because I didn’t like what was to offer and there was no alternative that I liked”* [009] and *“I was a finicky eater and then I’d kind of eat once I discovered what I felt I could eat.”* [022].

*“I’m really into like having orange juice like slushied*,* so like*,* I’ll freeze the orange juice too. Gets like a lot of consistency. But I know it takes exactly 3 hours. And so if I don’t have orange juice ready*,* I will wait until I have that orange juice. I’ll wait for it to slushify before I eat.”* [009].

Together, these factors often resulted in eating only when extremely hungry, increasing the risk of overeating and triggering binges.

*“I think that when I’m hyper-focusing I forget everything else*,* so I don’t eat or take care of myself. And so*,* I mess up my eating routine and then I’ll binge to “make up” for what I didn’t eat.”* [011].

Some of these factors also contributed to bingeing in the moment, such as interoceptive issues leading to mistaking thirst for hunger, missing fullness cues, fixating on certain foods that prompted overeating, or bingeing to avoid having to do shopping or meal preparation, saying *“I think something that led to bingeing is also not recognising that I’m full. […] I think my whole sense of like interoception is not great”* [001].

### Theme 3: binging to self-regulate

Participants noted that “*[bingeing] was probably like sensory seeking behaviour*” [002], with eating having “*become a bit of a sensory thing*” [023]. Participants described craving specific textural sensations like “*crunchy foods*” [001], or eating “*wholemeal bread and butter*,* like slice after slice*,* because I really liked the contrast of like the texture*” [002]. Participants also described craving physical sensations: “*swallowing specifically or like just having a feeling in my mouth*” [032], or the “*sensation of chewing*” [001].

Participants noted that alternatives like a “*heavy blanket or fidget toys*” [001], or choosing healthier foods with the same textures, could reduce loss of control and provide regulatory support, “*recognising that I just want the sensory input and I’m not actually hungry*,* helped me to choose more vegetables*” [001].

Stimulation seeking also shaped eating behaviours. Food was described as a way of “*procrastinating*” [032] or avoiding tasks, to cope with “*boredom*” [002], or when seeking a “*boost of dopamine*” [002]. One participant described strategies for obtaining this, including exercise or ‘dopamine menus’ of quick and pleasurable activities.

“*I’m struggling to do XYZ task. Maybe I need a little shot of dopamine before I do the task […] So that might be like crisps or drinks and really icy cold*,* sparkly water. Or sing my favourite song really*,* really loudly. Or run on the spot really hard for 30 seconds […] whatever it is*,* that just gives you like that boost of dopamine […] I just need something that I’m trying to put off this task or I’m trying to like get the task initiation*” [002].

Emotional regulation was another key function of food. Difficulties with emotional awareness, including “*recognising emotions*” [030] or “*acknowledging what they are*” [031], were seen as contributing to reliance on food for regulation. Food was also used as a form of self “*reward*” [030], a tool for “*relaxing*” [001], or as “*comfort*” [032], shifting its role beyond nutrition: “*food is pleasure*” [026], and “*I’m just waiting to be home […] where I could just relax*,* breathe*,* and don’t have to worry about the people around me and have my food in peace*” [009].

Finally, impulsivity and compulsive behaviours were described as amplifying binge behaviour, with participants reporting eating without conscious thought. This was described as “*formulaic*” [002] or as being on “*autopilot*” [022].

“*The impulsiveness*,* yeah. I just do it*,* I don’t think*,* I just do it and then when I’m doing it*,* that’s when I think ‘Oh*, ****’ […] And I’ve been known to travel far and wide to find a specific cake that I want. Umm,* you know*,* I’ve travelled across London to get a particular cake.*” [031].

### Theme 4: personalised and adaptable support

When discussing treatment preferences, many participants described a desire for care that was “*personalised*” [030] and adaptable to changing needs. As one participant explained, “*Neurodivergent people have different needs [that] might change over time*” [001]. Additionally, participants highlighted the importance of recognising the subjectivity of binges and noted that “*sensory aspects*” [001] of food should be considered within dietary advice.

Participants identified the need to accommodate challenges with “*abstract thinking*” in the therapy context [009], suggesting that more detailed and concrete approaches would be beneficial. Communication was described as most effective when it was clear, direct, and specific. For example, participants valued the use of “*specific questions”* [001] and emphasised the importance of consistent appointment times that *“don’t change”* [001].

Regarding structure, *“short*,* regular*,* and interactive sessions*” [011], were appreciated to help maintain engagement. Participants also reflected on the need for clinicians to remain mindful of Neurodivergence-related challenges, particularly around “*time perception*” [001] and “*memory*” [030], which may further shape eating behaviours and treatment engagement.

When reflecting on therapeutic options, one participant stated, “*I do think CBT can be super helpful for some Neurodivergent people because it’s very structured*” [001]. However, combining the structure of CBT with “*talking therapies [that] are generally more helpful*” [001] could be particularly effective, especially for individuals with ADHD who process verbally and may need more time to express themselves, “*I have ADHD and I’m a verbal processor*,* I’m Dyslexic you know all of these impacts how I process things*” [001].

Most participants had experience with individual therapy; however, when discussing how ED treatments could be improved for Neurodivergence individuals, they noted “*I think possibly having a community to turn to of people going through similar struggles would be nice*” [011].

In summary, participants valued ED services that offered personalised, flexible care while maintaining consistency and structure. Effective communication, consideration of sensory and cognitive differences, and the integration of both individual and community-based support were seen as central to meeting the needs of Neurodivergent individuals in ED treatment.

## Discussion

This study aimed to explore the experiences and treatment needs of Neurodivergent participants with BED, finding that Neurodivergence significantly shapes these experiences and needs. While some participants were uncertain about their Neurodivergence and its interaction with their ED, those with formal diagnoses reported greater self-understanding, helping them identify triggers and prevent binges. However, insights often came from social media rather than clinicians. ADHD-related factors such as missing hunger cues, sensory and executive challenges, and strong food preferences disrupted eating routines, increasing vulnerability to bingeing. Binge eating was also used to regulate sensory, stimulation, or emotional needs, with impulsivity intensifying this behaviour. Coping strategies included external prompts, alternative sensory inputs, exercise, and ‘dopamine menus.’ Participants stressed the importance of personalised, adaptable support that accounts for cognitive, sensory, and communication differences while fostering peer community.

### Perceived causal mechanisms

Our aim for this study was to expand the existing literature on Neurodivergence and EDs to include BED. Table 1 summarises prior qualitative findings on how Neurodivergent-related differences contribute to EDs from patients’ perspectives, with findings from this current study incorporated where relevant.

In our BED sample, similar mechanisms to those found in AN [8], included heightened exteroception leading to irregular or restrictive eating to avoid aversive sensations [14, 15, 17–19], and reduced interoception leading to skipped meals. This was somewhat unexpected, as compensatory behaviours are less common in BED [4], so we didn’t anticipate these findings. However, in BED, reduced interoception also contributed to bingeing by limiting awareness of fullness cues [14, 15, 17–19]. Both exteroceptive and interoceptive differences were also reported in BN [26]. Nonetheless, interoceptive differences, sensory avoidance, and preference for sameness were also present in our BED sample and were linked with irregular eating, though this was described as preceding eventual bingeing.

**Table 1:** Areas of convergence and divergence in qualitative findings on Neurodivergent-related differences contributing to EDs from lived experience perspectives

Neurodivergence-related differences	Description of difference	Behavioural outcome	Relevant samples
Exteroceptive hypersensitivity	Heightened external sensitivity to food, textures, tastes, smells, and environments	Restrictive or irregular eating to avoid aversive sensations	Mainly Autism-related, but also seen in ADHD, and across AN, BN, and BED
Exteroceptive hyposensitivity	Reduced external sensitivity to food, textures, tastes, smells, and environments	Bingeing for sensory input or to relieve under-stimulation	Mainly ADHD-related, and seen in BN and BED
Interoceptive hyposensitivity	Reduced internal sensitivity to hunger, thirst, digestion, or fullness	Accidental undereating or meal skipping; In BED, bingeing from not registering fullness cues	Mainly Autism-related, but also seen in ADHD, and across AN, BN, and BED
Emotions	Difficulties identifying, regulating, and communicating emotions, heightened emotions, and feelings of overwhelm	Restrictive eating to numb or distract from emotions; Bingeing to comfort or distract	Across both ADHD and Autism and in AN, BN, and BED
Monotropism	Focused interests and preference for routine	Strict rules around eating and food	Mainly Autism-related, and in AN and BN
Executive functioning	Hyperfocus, time-blindness, memory, and planning difficulties	Obsessional thoughts about bingeing; irregular meals due to distraction	Mainly ADHD-related, and seen in BN and BED
Social	Feeling different from others	Controlling weight to try to feel accepted by others	Mainly Autism-related, and in AN and BN

The emotional differences in our sample were also common across AN and BN, but their expression varied: in AN, patients reported *restricting* to numb or distract themselves [14, 15, 18, 19], whereas in BN and BED, patients mostly described *bingeing* to comfort or distract themselves [26]. Like in BN, participants with BED also reported bingeing for sensory input or relief from under-stimulation, alongside executive functioning differences that contributed to obsessional thoughts about food and irregular meals due to distraction [26]. The key differences in BED, compared to AN and BN, were the absence of thinking styles that drove strict food rules [14, 15, 17, 18, 26], and the absence of social differences and using weight control to seek acceptance from others [14, 15, 18, 19, 26].

**Fig. 2:**
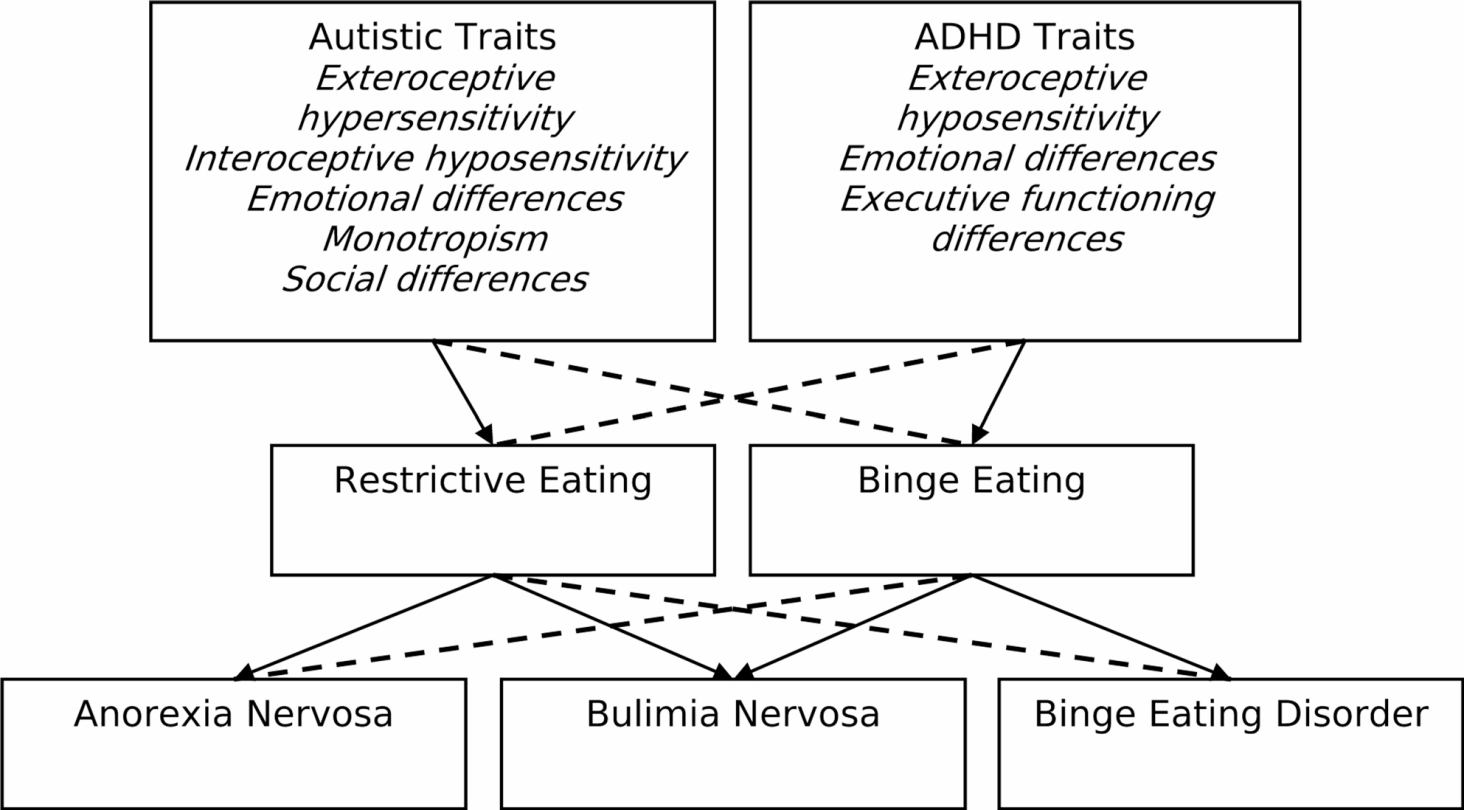
Transdiagnostic model on the contribution of Neurodivergence to EDs

Some of these differences may reflect the distinct development and presentation of BED compared to AN and BN. Our sample also included a lower proportion of Autistic participants than previous studies [8, 26], suggesting that certain differences may relate to the types of Neurodivergence represented. These two factors are likely connected, given that Autism is more commonly associated with restrictive EDs and ADHD with binge-type EDs [7]. Taken together, this supports a model (Fig. 2) in which Autistic traits contribute primarily (though not exclusively) to restrictive eating, while ADHD traits contribute primarily (though not exclusively) to binge eating. Thus, Autistic traits such as exteroceptive hypersensitivity, interoceptive hyposensitivity, emotional differences, monotropism, and social differences are more closely linked to AN and BN, whereas ADHD traits such as exteroceptive hyposensitivity, emotional differences, and executive functioning differences are more associated with BN and BED. Further research is needed to quantitatively validate and refine these relationships.

### Treatment needs

This study identified a series of treatment needs among Neurodivergent patients with BED, which mirrored or complemented those reported previously by Neurodivergent patients with AN and BN [8, 26], as shown in Table 2. These included: recognising Neurodivergence through screening and attentiveness to potential signs [14, 15, 18, 19, 26, 67]; delivering personalised treatments that account for both individual differences and differences from Neurotypical patients [14, 16–18, 26]; implementing sensory adaptions to dietary guidance, meal plans, and therapeutic environments, while also providing practical strategies for patients to use at home [14, 17, 18, 26]; communication adaptions such as offering varied methods and using clear, concrete language [14, 17–19]; maintaining consistent appointments and reminders, supporting the redirection of ED routines to non-food activities, and fostering sustainable, healthy routines, including regular meals [14, 17, 18, 26]; and allowing sufficient time to build therapeutic trust, facilitate processing and behavioural change, incorporate exploration of Neurodivergence within sessions, and provide regular breaks or shorter sessions if needed [17, 18, 26].

Interestingly, there were some needs highlighted in previous work that few patients in the current study raised, such as differentiating the ED from Neurodivergence and addressing traits only when they exacerbate the ED [17, 18, 26]; support for developing emotional and social skills and other coping skills [14–18]; or the need for staff training in Neurodivergence [17, 18]. This may partly reflect the higher proportion of participants who had never received ED treatment in our sample, as well as the lesser role of emotional and especially social differences in this group. This may also reflect that social skills trainings are often objected to by the Autistic community due to encouraging masking or self-stigmatisation if not implemented in a neuro-affirmative way [33].

**Table 2:** Summary of Neurodivergent-related ED treatment needs from lived experience perspectives

Treatment needs	Description	Studies
Recognise Neurodivergence	Implement screening and remain attentive to potential signs of Neurodivergence in patients	[14, 15, 18, 19, 26]*
Neuro-affirmative approach	Differentiate the ED from Neurodivergence and only address Neurodivergent traits if they worsen the ED, using gradual, Neuro-affirmative strategies.	[17, 18, 26]
Personalised treatment	Recognise individual differences among Neurodivergent patients and adapt treatment accordingly	[14, 16–18, 26]*
Sensory adaptions	Adjust dietary guidance, meal plans, and therapeutic environments to accommodate sensory needs, and provide practical strategies for patients to implement these adaptations at home	[14, 17, 18, 26]*
Communication adaptions	Offer varied communication methods, allow additional time for verbal processing, and use clear, concrete language while avoiding unnecessary abstraction	[14, 17–19]*
Consistency and routine	Maintain consistent appointments and reminders, support shifting ED routines to non-food activities, and promote sustainable, healthy routines including regular meals	[14, 17, 18, 26]*
Time and trust	Allow sufficient time to: build therapeutic trust, facilitate processing and behavioural change, incorporate exploration of Neurodivergence within sessions, and enable regular breaks or shorter sessions	[17, 18, 26]*
Emotional and social skills	Teach and support the development of adaptive coping strategies around managing emotional and interpersonal challenges within a neuro-affirmative framework	[14–18]
Staff training	Staff need adequate training on Neurodivergence to be able to implement all the above strategies	[17, 18]

*Plus the current study

Our findings highlight a need for ED services to better identify and support Neurodivergent patients, with space to explore the interplay between Neurodivergence and ED symptoms [14, 15, 17–19, 26]. Greater understanding of an individual’s Neurodivergence allows for more targeted strategies: For sensory needs, options include sensory workshops [68], DIY sensory toolkits, and interoception-based interventions like Aligning Dimensions of Interoceptive Experience (ADIE), which improves interoceptive awareness and reduces anxiety in Autistic adults [69]; and for emotional intensity, interventions may focus on acceptance-based CBT skills (shown to reduce negative urgency in binge-type EDs [70]), emotion wheels, structured meals, and alternative self-soothing strategies. Participants themselves emphasised the need for external prompts (e.g. timers, routines, or reminders) and pre-prepped food, to encourage regular eating; alternative sensory options (fidget toys, weighted blankets, or textured foods) to regulate sensory needs; and ‘dopamine menus’ to regulate stimulation needs [71].

Participants stressed the importance of personalised, adaptable support that accounts for cognitive, sensory, and communication differences, delivered by clinicians trained in Neurodivergence [14, 16–19, 26]. Prior studies note that Neurodivergent patients often feel misunderstood or harmed by generic CBT models [72]. Our findings reinforce the importance of tailored interventions, with clinicians recognizing how Neurodivergence affects therapy engagement and adapting accordingly (e.g., allowing fidget toys, avoiding abstract tasks, considering sensory needs when giving dietic advice), as recommended in other contexts [14, 17–19, 26, 73].

Initiatives like the PEACE Pathway [34], developed primarily for Autism and AN, already incorporate strategies like communication passports and sensory adaptations [35, 37, 38] that may also benefit other Neurodivergent ED populations. However, ADHD- and BED-specific adaptions remain underdeveloped. For instance, ADHD screeners such as the ASRS-Screener are not yet routinely implemented (though this may be changing [74]). There is a pressing need for accessible resources for both clinicians and patients that explain how Neurodivergence and related traits influence specific ED presentations and provide clear, evidence-based guidance for adapting treatment, offering practical templates to improve care consistency and outcomes.

### Strengths and limitations

This study is the first study to capture the patient perspectives of Neurodivergent individuals with BED, using inclusive recruitment, accessible interviews, and member reflections to address the double empathy problem. Conducted in an NHS service with an ethnically diverse sample, findings offer actionable insights for personalising treatment and improving engagement. Limitations include possible dilution from undiagnosed or subclinical participants; inability to compare neurotypes due to small subgroups; potentially limited transferability from the small, self-selecting sample; and potential bias towards those engaged with services or interested in Neurodivergence.

## Conclusions

Neurodivergent traits contribute transdiagnostically to EDs, and ED services should try to support patients and clinicians in recognising and understanding Neurodivergence. Clinicians need training to identify how Neurodivergent differences around sensory processing, attention, executive functioning, and emotion contribute to BED. Treatment should address these mechanisms, providing flexible, individualised care.

## Data Availability

The datasets used during the current study are available from the corresponding author on reasonable request and can be used for secondary analysis as is stipulated in participants’ consent forms.

## Abbreviations

α: Cronbach’s alpha
AASPIRE: Academic Autism Spectrum Partnership in Research and Education
ADHD: Attention-deficit/hyperactivity disorder
ADIE: Aligning Dimensions of Interoceptive Experience
AN: Anorexia nervosa
AQ-10: Autism Spectrum Quotient – 10-item
ASRS-Screener: Adult ADHD Self-Report Scale Screener
BED: Binge eating disorder
BMI: Body Mass Index
BN: Bulimia Nervosa
CAT: Cognitive Analytic Therapy
CBT-E: Enhanced Cognitive Behavioural Therapy
CBT-T: 10-Session Cognitive Behavioural Therapy
DSM-IV-TR: Diagnostic and Statistical Manual of Mental Disorders, Fourth Edition, Text Revision
ED: Eating disorder
GSQ-14: Glasgow Sensory Questionnaire – 14-item
M: Mean
n: Number of Participants
NHS: National Health Service
NICE: National Institute for Health and Care Excellence
OSFED: Other Specified Feeding or Eating Disorder
PEACE: Pathway for eating disorders and autism developed from clinical experience
PhD: Doctor of Philosophy
PTSD: Post-Traumatic Stress Disorder
RTA: Reflexive thematic analysis
SLaM: South London and Maudsley
